# Emergence of Dendritic Cells in the Myocardium after Acute Myocardial Infarction – Implications for Inflammatory Myocardial Damage

**Published:** 2010-03

**Authors:** Atilla Yilmaz, Barbara Dietel, Iwona Cicha, Katja Schubert, Roland Hausmann, Werner G. Daniel, Christoph D. Garlichs, Christian Stumpf

**Affiliations:** 1 *Clinic of Internal Medicine I, Department of Cardiology, University Hospital Jena, Jena, Germany;*; 2 *Medical Clinic II, University Hospital Erlangen, Erlangen, Germany;*; 3 *Department of Forensic Medicine, University Basel, Switzerland*

**Keywords:** adaptive immunity, atherosclerosis, innate immunity, leukocyte, dendritic cells

## Abstract

Dendritic cells (DC) are crucial for T cell mediated immune responses. Recently, we observed a significant decrease in circulating myeloid DC precursors in patients with acute myocardial infarction (AMI). The aim of the present study was to investigate whether myeloid DC are present in infarcted myocardium. Myocardial specimens of 10 patients with AMI and 7 accident victims (controls) were collected after autopsy. In immunostainings the presence of DC (CD209^+^, fascin^+^), T cells (CD3^+^), macrophages (CD68^+^), and HLA-DR expression was analyzed. Significantly higher numbers of CD209^+^-DC (97 vs. 44 cells/0.25 mm^2^, p=0.03), fascin^+^-DC (54 vs. 8 cells/0.25 mm^2^, p=0.02), T cells (27 vs. 6 cells/0.25 mm^2^, p=0.02), and macrophages (44 vs. 6 cells/0.25 mm^2^, p=0.01) associated with high HLA-DR expression were detected in infarcted myocardium. Frequent colocalizations of DC and T cells were observed. In occluded coronary arteries numerous DC, T cells, macrophages and high HLA-DR expression were found. We show that DC are present in infarcted myocardium after AMI. High HLA-DR expression and the colocalization with T cells suggest that they might trigger an immune response leading to further myocardial damage.

## INTRODUCTION

The initial inflammatory response after acute myocardial inflammation (AMI) is a prerequisite for healing and scar formation, whereas overwhelming and persisting myocardial inflammation is responsible for further destruction of vital myocardium promoting left ventricular (LV) remodelling and consecutively heart failure (1). Correspondingly, it was shown that elevated serum levels of pentraxin-3, high sensitivity (hs)CRP, and amyloid a are independent predictors of a worse outcome after AMI (2–4). However, therapeutic interventions using corticosteroids or nonsteroidal anti-inflammatory drugs to suppress myocardial inflammation failed to be effective to prevent LV remodelling after AMI, and were even associated with a higher incidence of infarct expansion and cardiac rupture (5, 6).

Beyond acute inflammation, it was described that immune reactions against cardiac contractile proteins such as actin, myosin, or troponin emerge after AMI, leading to persisting myocardial inflammation, which promotes LV remodelling and progressive heart failure (7, 8) Further characterization of that immune reaction showed that the presence of Th1 cells is associated with a worse clinical outcome (9). In concordance with that, it was shown in animal models that suppression of the invasion of immune cells into the infarcted myocardium had a protective effect on LV remodelling after AMI (10, 11). These results suggest that autoimmunity may contribute to further myocardial damage after AMI.

Dendritic cells (DC) are potent antigen-presenting cells, required for the induction of each immune response through antigen-specific activation of T cells (12). In various autoimmune diseases, DC play a crucial role, inducing an immune response against self antigens (13). In atherosclerosis, it was assumed that DC contribute to progression and destabilization of atherosclerotic plaques (14). Proving this concept, Han *et al*. recently demonstrated in a vascular model that DC are the most potent T cell activators in atherosclerosis and are pivotal for the immune reactions in the vessel wall microenvironment (15).

In two recent studies, it was described that circulating DC precursors are significantly, transiently reduced in acute coronary syndromes (ACS) (16, 17). It was suggested that they might be recruited into the atherosclerotic vascular wall (16). However, the vast difference in the levels of circulating myeloid DC precursors between patients with unstable coronary artery disease (CAD) and those with ST segment elevation-AMI made it very likely that they might be additionally recruited into the infarcted myocardium (16).

Therefore, the aim of our present study was to investigate whether DC are present in the infarcted myocardium of patients with AMI, and if they are able to trigger there an immune response through antigen-specific activation of T cells.

## METHODS

### Patients and controls

**Figure 1:**
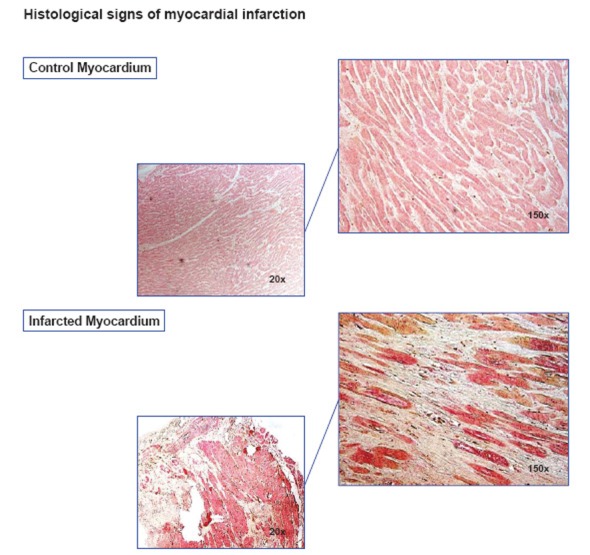
Morphology of healthy and infarcted myocardium. The area of infarction was histologically assessed using a haematoxylin/eosin staining. Upper panel: Healthy myocardium of an accident victim. Lower panel: Myocardium of an individual with acute myocardial infarction with typical histological signs, e.g. hyerpeosinophilia, alteration of striation, loss of nuclei, and oedema.

In collaboration with the Department of Forensic Medicine of the University Hospital Erlangen myocardial specimens were collected from 10 individuals with AMI deceased after sudden cardiac death and 7 individuals after fatal accident as controls. Myocardial specimens were collected from the macroscopically evident infarction area and from corresponding areas of the controls. Histological analyses were performed using a haematoxylin/eosin staining (Figure 1). The diagnosis of an AMI was confirmed by macroscopical and microscopical evaluation, as described (18). Since patients died before admission in the hospital, we were not able to record clinical data. The study was carried out in accordance with the Declaration of Helsinki of the World Medical Association (19).

### Immunohistochemical Analysis

**Figure 2:**
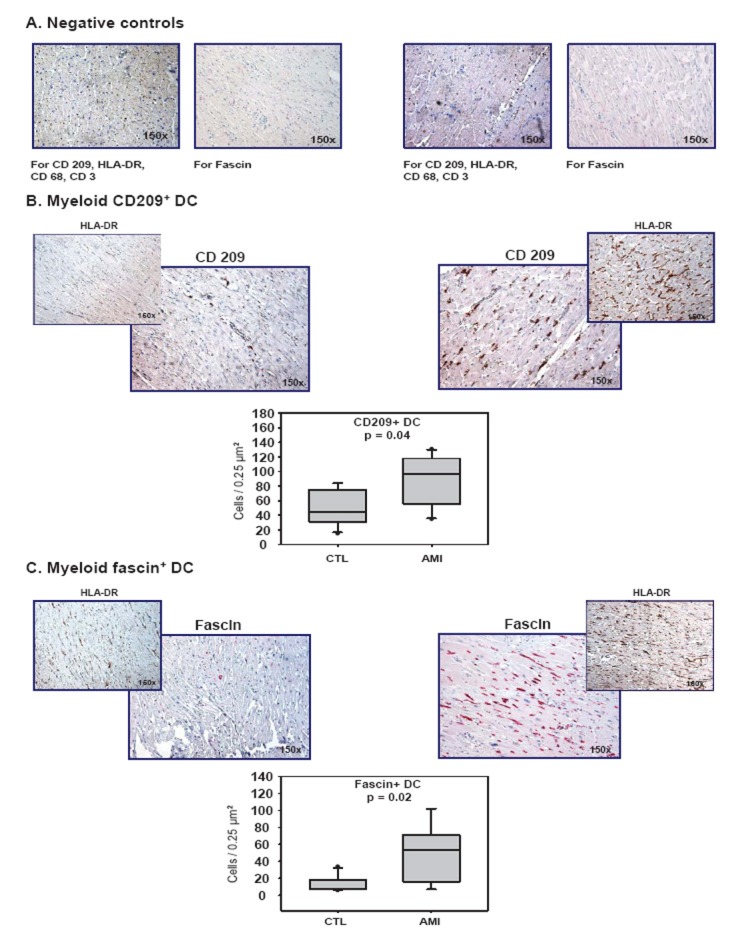
Emergence of myeloid DC in infarcted myocardium. **(A)** Negative (isotype) controls for CD 209, HLA-DR, CD 3, and CD 68 (CSA immunostaining) as well as for fascin (Envision G/2 immunostaining) in control (left) and infarcted myocardium (right). **(B, C)** Identification of DC by the specific markers CD 209 (CSA, Becton Dickinson 1:50) and fascin (Envision G/2, Dako, 1:100), and corresponding expression of HLA-DR (CSA, Dako, 1:25) in control (left) and infarcted myocardium (right). Histographical presentation: box plot of the number of CD 209^+^- and fascin^+^-DC (cells/0.25 µm^2^) in control (CTL) and infarcted (AMI) myocardium. Statistical analysis by *Mann-Whitney Rank Sum Test.*

For immunohistochemical staining, the following monoclonal antibodies were used: anti-CD209 (1:50; Becton Dickinson, Heidelberg, Germany) for immature and anti-fascin (1:100; Dako, Hamburg, Germany) for mature myeloid DC, anti-CD3 (1:80; Dako, Hamburg, Germany) for T cells, anti-CD68 (prediluted, Dako) for macrophages, and anti-HLA-DR (1:25; Dako). For immunohistochemical stainings of CD209, CD3, CD68, and HLA-DR the *Catalyzed Signal Amplification kit*™ (Dako) and for fascin staining the *EnVision G/2 Detection System*™(Dako) were usedaccording to manufacturer’s instructions. Double immunohistochemical staining was performed using the EnVisionTM Doublestain System (Dako) according to manufacturer’s instructions. Negative controls for single or double immunostainings were treated with isotype-matched antibodies (Figure 2A, 5A).

Stained cells were counted in the infarction area of AMI patients with a CCD-camera (Nikon DXM 1200, Düsseldorf, Germany) at a magnification of 150x in each five representative sections (each 0.25 µm^2^). For controls, corresponding areas were analyzed. For each patient, median cell numbers of CD209^+^- and fascin^+^-DC, CD68^+^-macrophages, CD3^+^-T cells, and HLA-DR^+^-cells in the myocardium were calculated.

### Statistical analysis

All values are reported as median. P<0.05 was considered statistically significant. The non-parametric *Mann-Whitney Rank Sum Test* was used to compare the median number of different immune cells between AMI patients and controls. Correlation analyses were performed using the *Spearman Rank Order Test*.

## RESULTS

### Emergence of DC, T cells and macrophages in infarcted myocardium

In the present study, we investigated if myeloid DC, which were shown to be significantly reduced in the circulation of patients with AMI, might be present in infarcted myocardium of patients deceased after sudden cardiac death. All patients in the AMI group had macroscopically and microscopically evident signs of myocardial infarction (Figure 1).

**Figure 3:**
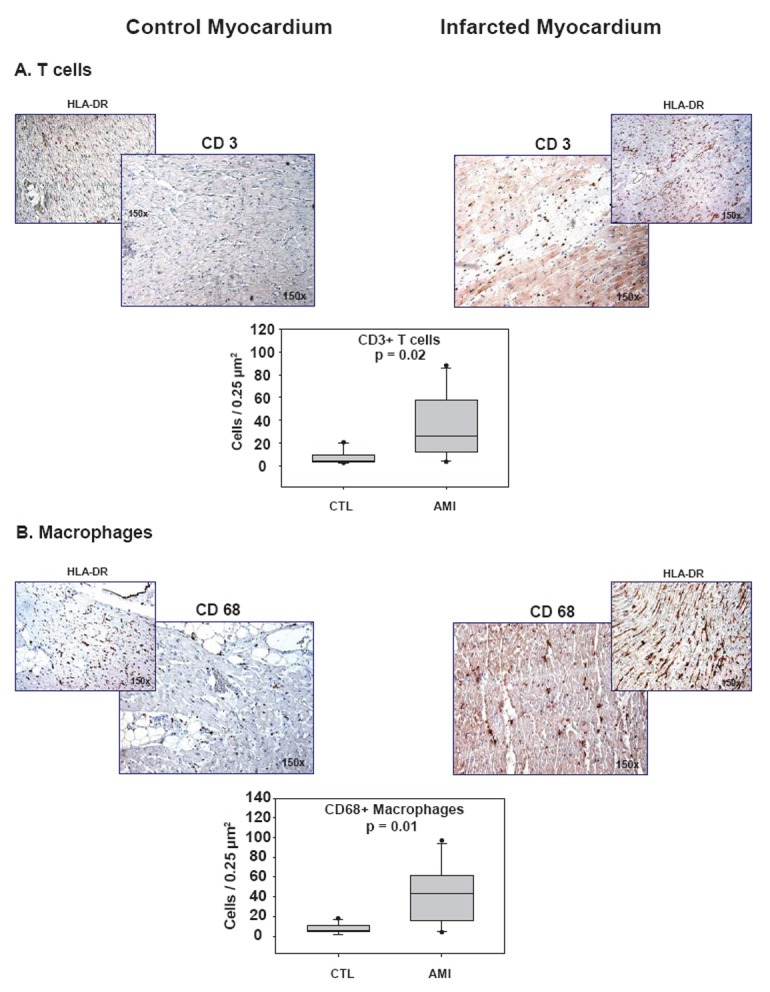
Presence of T cells and macrophages in infarcted myocardium. Images of immunostainings of **(A)** T cells (CSA, CD3^+^, Dako, 1:80) and **(B)** macrophages (CSA, Dako, CD68^+^, prediluted) together with HLA-DR expression (CSA, 1:25) in control (CTL) and infarcted (AMI) myocardium and corresponding histographical presentation. Statistical analysis by *Mann-Whitney Rank Sum Test*

In infarcted myocardium, we observed significantly higher numbers of DC, detected by their specific expression of CD209 (97 vs. 44 cells / 0.25 mm^2^, p = 0.03, Figure 2B) or fascin (54 vs. 8 cells / 0.25 mm^2^, p = 0.02, Figure 2C), compared to healthy control myocardium. Furthermore, significantly more T cells (27 vs. 6 cells / 0.25 mm^2^, p = 0.02, Figure 3A) and macrophages (44 vs. 6 cells / 0.25 mm^2^, P = 0.01, Figure 3B) were observed in infarcted than in control myocardium. Associated with the presence of DC, macrophages, and T cells was a strong expression of HLA-DR (Figure 2 and 3).

### Significant correlation between different types of immune cells and HLA-DR

**Figure 4:**
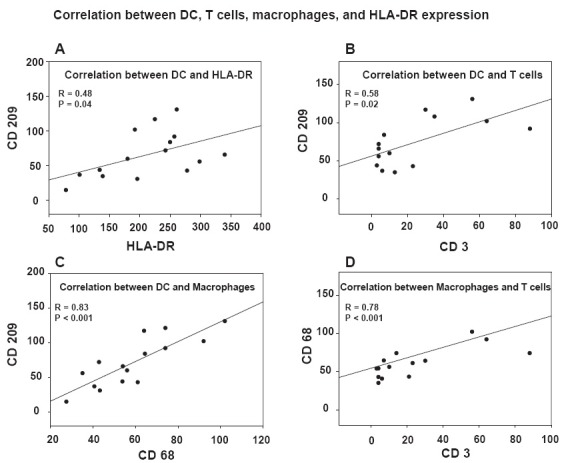
Significant correlation between different types of immune cells and HLA-DR expression. Correlation analyses (*Spearman Rank Order Test*) were performed comparing the number of DC, T cells, macrophages, and HLA-DR^+^ cells to each other, which were evaluated by immunohistochemical stainings and subsequent digital image analysis. **(A)** Correlation between CD209^+^-DC and HLA-DR^+^-cells, **(B)** between CD209^+^-DC and CD3^+^-T cells, **(C)** between CD69^+^-macrophages and CD209^+^-DC, and **(D)** between CD69^+^-macrophages and CD3^+^-T cells. On both axis, the number of cells per 0.25 µm^2^ is indicated.

The emergence of different types of immune cells and HLA-DR expression in the infarcted myocardium was compared by correlation analysis. We show a significant correlation between CD209^+^-DC and HLA-DR-positive cells (r = 0.48, p = 0.04, Figure 4A). Additionally, a significant correlation between DC and T cells (r = 0.58, p = 0.02, Figure 4B) was observed. These results suggest that DC significantly contribute to HLA-DR expression and might be colocalized to T cells, so that all preconditions are fulfilled which are necessary for the induction of a DC-mediated immune response.

Furthermore, a significant correlation between DC and macrophages (r = 0.83, p < 0.001, Figure 4C) was detected, which is not surprising, since both myeloid DC and macrophages are monocyte-derived cells with a similar migratory pattern in response to inflammatory stimuli. Similar to DC, macrophages significantly correlated with T cells (r = 0.78, p < 0.001, Figure 4D), suggesting that they might contribute to T cell activation too.

### Colocalization of DC with T cells

**Figure 5:**
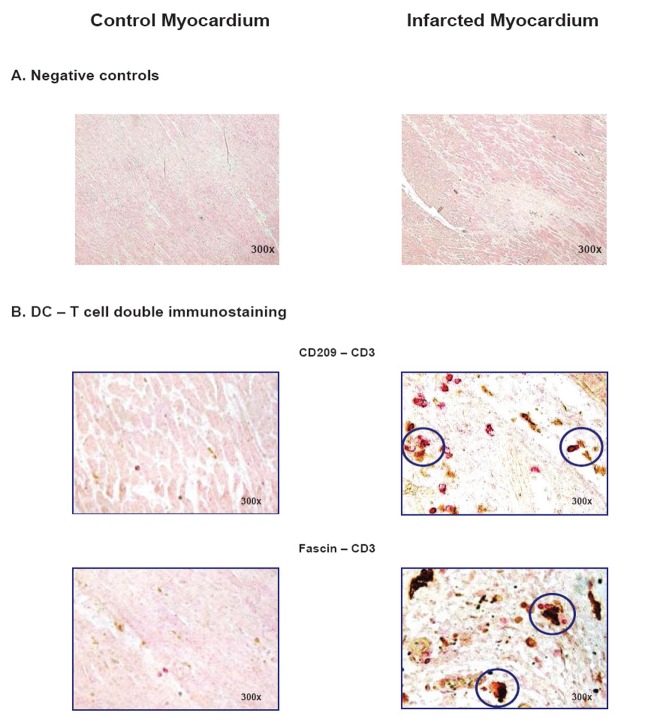
Cell contacts between DC and T cells. Double immunohistochemical stainings (Envision TM Doublestain system, Dako) of CD209^+^-DC (brown, 1:50) vs. CD3^+^-T cells (red, 1:80) and fascin^+^-DC (brown, 1:100) vs. CD3^+^-T cells (red, 1:80) were performed to show cell contacts between DC and T cells. **(A)** Negative (isotype) controls and **(B)** control myocardium (left) compared to infarcted myocardium (right). Direct cell contacts are indicated by circles.

Beyond correlation analysis, the colocalization of DC with T cells was investigated in a more direct manner using double immunohistochemical staining. The proof of a colocalization of DC and T cells is pivotal, since the induction of a DC-initiated immune response is only possible, when both cell types have close physical contacts. In contrast to control, we were able to show numerous contacts between DC (CD209^+^ or fascin^+^) and T cells (CD3^+^) in infarcted myocardium, which exceeded by far the number of cell contacts expected by random distribution (Figure 5B). Hence, these results appear most consistent with the interpretation that DC may be directly activating T cells in infarcted myocardium.

### Emergence of different immune cells and HLA-DR in occluded coronary arteries

**Figure 6:**
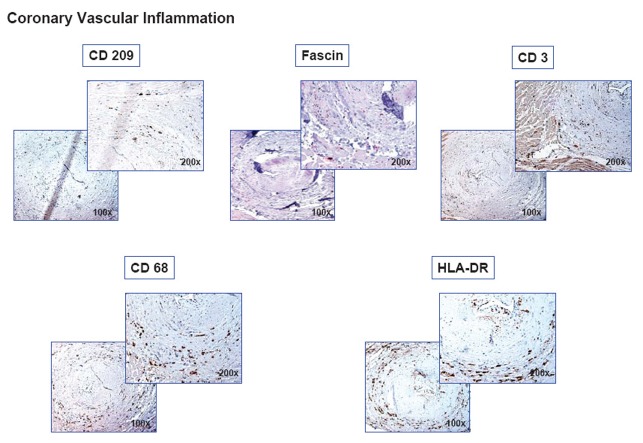
Emergence of different immune cells and HLA-DR in occluded coronaries. Immunohistochemical stainings (conditions see Figure 2) of CD209^+^-DC, fascin^+^-DC, CD3^+^-T cells, CD69^+^-macrophages, and high HLA-DR expression in a thrombotically occluded intramyocardial coronary artery.

Frequently, coronary arteries with thrombotical occlusions were observed in the myocardial specimens of patients with AMI. Compared to healthy vessels of controls, in thrombotically occluded coronaries a dense infiltration of all vascular layers with CD209^+^-DC, fascin^+^-DC, T cells, macrophages, and a high HLA-DR expression was observed (Figure 6), indicating a high coronary vascular inflammation. However, we were unable to determine whether coronary inflammation precedes, accompanies, or follows AMI.

## DISCUSSION

Beyond acute inflammation upon AMI, a persisting autoimmune response against cardiac proteins emerges, which is responsible for further myocardial damage leading to progressive heart failure (20). Hence, it was shown that adoptive transfer of splenocytes of post-AMI rats into syngeneic rats was associated with organ specific autoimmune myocarditis (21). Furthermore, in blood of humans after AMI the presence of autoantibodies and cellular autoimmunity against cardiac proteins was detected (7, 8). In this context, it was shown that in patients with AMI a Th1 response, present as high ratio of interferon-γ-producing blood T cells, persists over months. This enduring, overwhelming autoimmune response gets clinically apparent in some cases several weeks after AMI as *Dressler’s syndrome* (22).

DC play a pivotal role through initiating and modulating antigen-specific activation of T cells. It was shown that DC can be attracted into infarcted tissues such as brain, kidney and liver, and are able to induce an autoimmune response there (23–25). However, the role of DC in human AMI was unknown yet. Recently, we described that circulating myeloid DC precursors are significantly reduced in patients with AMI, indicating that they might be recruited into the infarcted myocardium (16). Thus, the aim of the present study was to investigate if myeloid DC might be present in human myocardium after AMI.

We show for the first time that DC infiltrate human infarcted myocardium of patients deceased after AMI. Considering the fact that circulating myeloid DC precursors are significantly, transiently reduced in patients with AMI (16), it is most likely that upon AMI circulating DC precursors are recruited into the infarcted myocardium. Corresponding to this hypothesis, two third of the DC found in infarcted myocardium expressed an immature phenotype (CD 209 expression) compared to one third mature DC (fascin expression). This result is explained by the fact that the analysis was performed at a very early stage of AMI, since patients died very soon after onset of symptoms from sudden cardiac death, so that a maturation of DC, which endures several days, was not possible in this case. However, technical reasons, e.g. different staining procedures, can not been completely excluded.

Furthermore, numerous T cells and macrophages were detected in the infarcted myocardium, and they significantly correlated with the number of DC. This fact is very important because single DC are not able to mediate effects of the immune system, but the presence of further immune cells is required like an orchestra, so that DC can initiate an immune response. In the area of infarction a strong expression of HLA-DR, which significantly correlated with the presence of DC, was observed. Furthermore, direct cell contacts of DC and T cells were detected, so that it is very likely that DC induce an immune response to cardiac antigens leading to myocardial inflammation after AMI.

These results are consistent with former animal studies analyzing the recruitment of DC into the infarcted myocardium. Zhang *et al*. firstly described the presence of DC, associated with MHC II expression and T cell contacts, in the heart of rats with experimental AMI (26). More recently, Naito *et al*. demonstrated that GM-CSF and G-CSF inversely affected LV remodelling after experimental AMI, and they concluded that modulation of the immune response by myocardium-infiltrating DC is crucial for the development of progressive heart failure (27). Additionally, Takahashi *et al*. showed that locally injected high-mobility group box (HMGB)-1 significantly reduced the myocardial infiltration of DC after AMI, and, thus, improved global cardiac function (28).

Beyond myocardial inflammation, we showed significant inflammation of occluded coronary arteries. However, coronary vascular inflammation might be the reason or the result of AMI, which can not be determined in the present study. Recently, it was shown that platelets induce vascular recruitment and maturation of DC (29). Hence, interaction of platelets with DC might be the link between thrombosis and coronary inflammation.

## CONCLUSIONS

The results of our study show that infarcted myocardium is significantly infiltrated with DC, macrophages, and T cells. The high expression of HLA-DR and frequent contacts with T cells indicate that DC initiate an immune response against cardiac antigens in the infarcted myocardium leading to progressive heart failure. Thus, modulation of DC function might be a novel therapeutic approach to prevent LV remodelling after AMI.
